# Positional and functional mapping of a neuroblastoma differentiation gene on chromosome 11

**DOI:** 10.1186/1471-2164-6-97

**Published:** 2005-07-06

**Authors:** Katleen De Preter, Jo Vandesompele, Björn Menten, Philippa Carr, Heike Fiegler, Anders Edsjö, Nigel P Carter, Nurten Yigit, Wim Waelput, Nadine Van Roy, Scott Bader, Sven Påhlman, Frank Speleman

**Affiliations:** 1 Center for Medical Genetics, Ghent University Hospital MRB 2^nd ^floor, De Pintelaan 185, B-9000 Ghent, Belgium; 2 The Wellcome Trust Sanger Institute, Wellcome Trust Genome Campus, Hinxton, Cambridge CB10 1SA, United Kingdom; 3 Department of Laboratory Medicine, Molecular Medicine, Lund University, University Hospital MAS, S-20502 Malmö, Sweden; 4 Department of Pathological Anatomy, Ghent University Hospital BLOK A, De Pintelaan 185, B-9000 Ghent, Belgium; 5 Sir Alastair Currie Cancer Research U.K. Laboratories, Division of Pathology, Molecular Medicine Centre, University of Edinburgh, Crewe Road, Edinburgh EH4 2XU, United Kingdom

## Abstract

**Background:**

Loss of chromosome 11q defines a subset of high-stage aggressive neuroblastomas. Deletions are typically large and mapping efforts have thus far not lead to a well defined consensus region, which hampers the identification of positional candidate tumour suppressor genes. In a previous study, functional evidence for a neuroblastoma suppressor gene on chromosome 11 was obtained through microcell mediated chromosome transfer, indicated by differentiation of neuroblastoma cells with loss of distal 11q upon introduction of chromosome 11. Interestingly, some of these microcell hybrid clones were shown to harbour deletions in the transferred chromosome 11. We decided to further exploit this model system as a means to identify candidate tumour suppressor or differentiation genes located on chromosome 11.

**Results:**

In a first step, we performed high-resolution arrayCGH DNA copy-number analysis in order to evaluate the chromosome 11 status in the hybrids. Several deletions in both parental and transferred chromosomes in the investigated microcell hybrids were observed. Subsequent correlation of these deletion events with the observed morphological changes lead to the delineation of three putative regions on chromosome 11: 11q25, 11p13->11p15.1 and 11p15.3, that may harbour the responsible differentiation gene.

**Conclusion:**

Using an available model system, we were able to put forward some candidate regions that may be involved in neuroblastoma. Additional studies will be required to clarify the putative role of the genes located in these chromosomal segments in the observed differentiation phenotype specifically or in neuroblastoma pathogenesis in general.

## Background

In addition to the well known group of high stage neuroblastomas with *MYCN *amplification and 1p-deletion, a second genetic subgroup of aggressive neuroblastomas has been delineated. This subgroup is characterised by the presence of 11q-deletions, often in association with 3p-deletions [1-5]. Both subgroups typically present with 17q-gain or a normal chromosome 17 copy number, which are the strongest independent genetic indicators of poor prognosis [6]. Deletions of 11q mostly affect a large distal part of the long arm. Only a few small deletions have been identified which delineated a tentative SRO (shortest region of overlap) at 11q23 between markers D11S1340 and D11S1299, encompassing a region of approximately 3 Mb [7]. More recently however, a neuroblastoma patient was reported with a constitutional 11q14.1-11q23.3 deletion that did not overlap with the proposed SRO [8]. Consequently, the presumed localisation of the 11q neuroblastoma tumour suppressor gene (or genes) remains ill defined, thus hampering the selection of positional candidate genes. For the 11q23 region we proposed *SDHD *as a putative candidate neuroblastoma tumour suppressor, but only two bona fide mutations could be identified[9].

In addition to the observed losses of 11q in neuroblastoma, the existence of a tumour suppressor gene on 11q has also been supported by functional evidence obtained by microcell mediated chromosome 11 transfer (MMCT) experiments [10]. Although these studies were initially aimed at investigating the role of chromosome 1p in tumour suppression, the control chromosome 11 transfer experiment unexpectedly produced clones with morphological features of differentiation. Introduction of chromosome 11 induced a more flattened and adherent morphology, with short neuritic processes, similar to the changes seen after a few days of growth in the presence of retinoic acid. As these microcell hybrids could be powerful models for the identification of candidate neuroblastoma suppressor or differentiation genes, we decided first to determine the genetic status of the chromosome 11 in the hybrid subclones prior to further experiments. To this purpose, the parental NGP cell line and the microcell hybrids after chromosome 11 transfer were analysed using high-resolution arrayCGH (microarray based comparative genomic hybridisation), FISH (fluorescence *in situ *hybridisation) and microsatellite heterozygosity mapping. Following the identification of a region on chromosome 11 with altered copy number, we measured the mRNA expression levels of genes in these regions in an attempt to find altered gene expression related to neurite outgrowth and differentiation.

## Results

### Morphological characterisation

**Figure 1:**
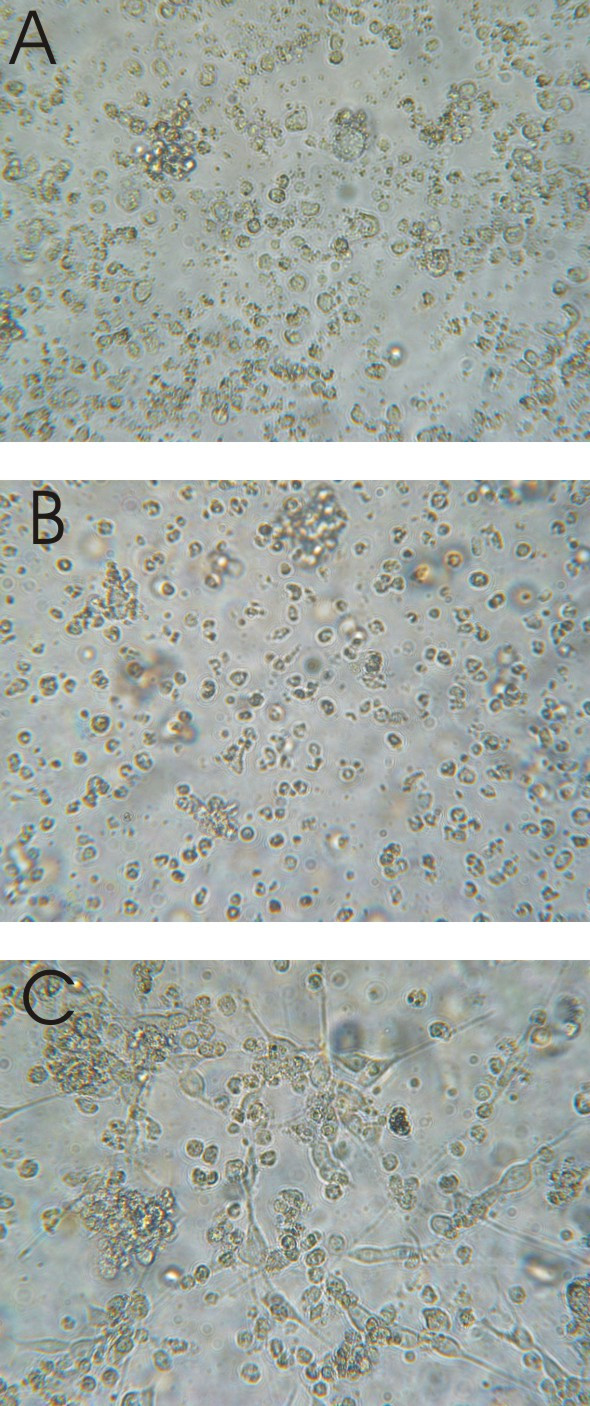
Cell morphology of parental cell line NGP.1A.TR1 (A) and chromosome 11 transferred subclone MCH574c3 (B) with non-adherent, spheroid cells, and subclone MCH574c10 (C) showing signs of induced differentiation such as short neuritic processes

The chromosome 11 status of the different microcell hybrid subclones used in this study and the reported chromosome 11 changes [10] are listed in Table 1. The morphology of the cells was comparable to the phenotype described by Bader and colleagues [10]. Cells of the parental cell line NGP.1A.TR1 (a tumour reconstitute of mutagenised NGP cells [10]) were non-adherent, spheroid and growing in cell clusters (Figure 1A). Subclones with an apparently intact transferred chromosome 11 (MCH574c4, c11, c13), as well as the clone with reported loss of a region on 11q (MCH574c10) exhibited features of induced differentiation, with more flattened and adherent cells and some short neuritic processes (Figure 1C). Subclone MCH574c3 with reported loss of part of 11p showed the same non-adherent phenotype as the parental cell line NGP.1A.TR1 (Figure 1B).

**Table 1:** Chromosome 11 status and morphology of the microcell hybrids (MCH) obtained after chromosome 11 transfer in parental NGP.1A.TR1 cells as determined by Bader and colleagues [10] and in this study

**microcell hybrid subclone (NGP.1A.TR1 + chr 11)**	**chromosome 11 status (in addition to parental NGP.1A.TR1 11q-loss)**		**morphology**
		
	**Bader et al. [10]**	**this study**	
MCH574c4,c11,c13	no additional changes	del(11)(pterp15.1)	more flattened, adherent cells, some short neuritic processes
MCH574c10	del(11)(q23.3) (MCT128.1, HBI 18P2)	del(11)(pterp15.1)	more flattened, adherent cells, some short neuritic processes
MCH574c3	del(11)(p15.5) (*HRAS*)	del(11)(pterp15.1) del(11)(pterp13) del(11)(q25qter)	non-adherent, spheroid cells, growing in cell clusters

Assessment of the organisation of the actin fibres using phalloidin staining confirmed the presence of neurites (and excluded stress fibres) in subclones MCH574c10 and MCH574c11[11].

### ArrayCGH based chromosome 11 copy number assessment

**Figure 2:**
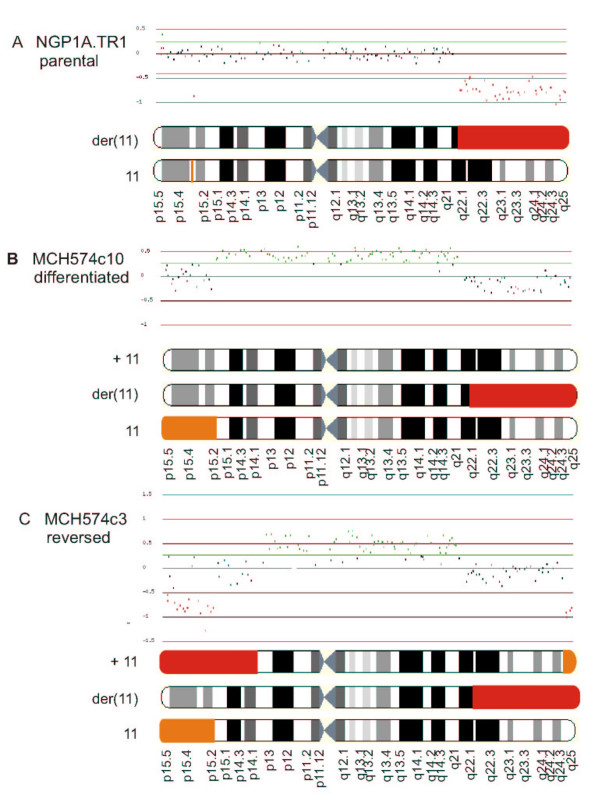
ArrayCGH results (log2 scale) of parental cell line NGP1A.TR1 and microcell hybrids MCH574c3 and MCH574c10 compared to a normal female control, with reported (red) and newly detected (orange) chromosome 11 deletion events, (A) parental cell line (NGP.1A.TR1), (B) MCH574c10 in which regional 11q-loss of the transferred chromosome 11 was reported [10] and (C) MCH574c3 with reported regional 11p-loss of transferred chromosome 11. FISH was used to confirm the results obtained by arrayCGH (data not shown).

ArrayCGH was performed for NGP.1A.TR1, MCH574c3 and MCH574c10 cells. These hybridisations failed to provide evidence for the reported 11q-deletion in the transferred chromosome of microcell hybrid MCH574c10 (Figure 2). Unexpectedly, the distal region of the short arm of one of the chromosomes 11 (11pter->11p15.1) was deleted in both MCH574c3 and MCH574c10. Microcell hybrid MCH574c3 presented with an additional larger deletion of 11pter->11p13, as well as a third deletion involving the most distal band (11q25->11qter) in one of the chromosomes 11. Deletion of a single BAC clone RP11-51B23 on 11p15.3 was detected in the parental NGP.1A.TR1 cells (Figure 2B). Thus far, this clone has not been recognised as being involved in polymorphic genomic deletions for this particular chromosomal region (own observations and Ensembl clone list). Deletions observed by arrayCGH were confirmed by FISH analysis using one BAC clone selected in each observed deleted region (RP11-734D5 on 11p15.3, RP11-48O9 on 11p13, RP11-545G16 on 11q25). This FISH analysis demonstrated that the 11pter->11p15.1 deletion was present in all other subclones that were not analysed with arrayCGH, i.e. MCH574c4, c11 and c13.

### Microsatellite heterozygosity mapping

To determine which of the chromosomes 11 exhibited loss of the 11pter->11p15.1, 11pter->11p13 and 11q25->11qter regions, microsatellite heterozygosity mapping in conjunction with FISH analysis of metaphase spreads was performed. Microsatellite markers D11S861 (on 11p15.2) and D11S1324 (on 11p14.1) were tested on NGP.1A.TR1, MCH574c3 and MCH574c10. These tests show that one of the two parental chromosomes 11 had lost the 11pter->11p15.1 region, while the 11pter->11p13 segment was lost in the transferred chromosome. FISH on metaphase spreads (clone RP11-545G16 on 11q25 in combination with RP11-206C1 on 11p15.1; clone RP11-709M17 on 11q25 in combination with clone RP11-4B7 on 11p15.2) demonstrated that the 11q25->11qter deletion occurred in the transferred chromosome 11, whereas the 11pter->11p15.1 deletion occurred in the normal parental chromosome 11 (and not in the parental der(11)t(2;11)) (Figure 2).

### Breakpoint delineation of chromosome 11 deletions

**Figure 3:**
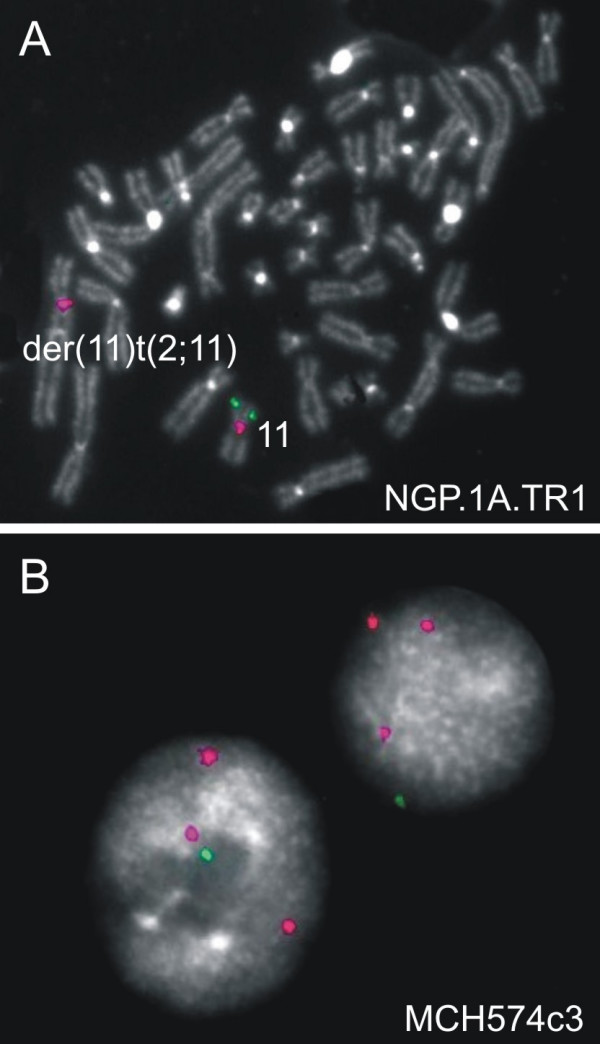
FISH analysis of BAC clones RP11-573E11 (panel A), RP11-51B23 and RP11-47J17 (not shown) in parental cells NGP.1A.TR1 (with a derivative chromosome 11, due to an unbalanced translocation between chromosomes 11 and 2) confirms the existence of a small deletion in 11p15.3. The breakpoint of the distal 11q25->11qter deletion of the transferred chromosome in subclone MCH574c3 was mapped between BAC clones RP11-340L13 (not shown) and RP11-697E14 (panel B).

The position of the deletion breakpoints was confirmed or refined by FISH analysis. The breakpoint of the del(11)(q22.1qter) resulting from an unbalanced translocation between chromosomes 2 and 11 in parental cell line NGP.1A.TR1 mapped within a 2.285 Mb segment located between BAC clones RP11-379J13 and RP11-49M9 (map position between 97.328 Mb and 99.613 Mb, NCBI 35 May 2004 assembly (hg17)). The breakpoint of the 11pter->11p15.1 deletion of the normal parental chromosome 11 in all microcell hybrids was assigned to a 229 kb segment between RP11-452G18 and RP11-358H18 (17.337 Mb – 17.596 Mb). The breakpoint of the larger 11p-deletion (11pter->11p13) present in MCH574c3 was located within a 921 kb segment flanked by clones RP11-48O9 and RP11-202M19 (33.038 Mb – 33.959 Mb). The breakpoint of the distal ± 13 Mb 11q25->11qter deletion of the transferred chromosome in subclone MCH574c3 was mapped between BAC clones RP11-340L13 and RP11-697E14 (131.149 Mb – 131.230 Mb). Flanking clones of the 11p15.3 deletion in NGP.1A.TR1 were also tested using FISH (RP11-734D5, RP11-573E11, RP11-47J17) demonstrating that the deletion involves at least a 707 kb segment including BAC clones RP11-573E11 and RP11-47J17 (12.202 Mb – 12.909 Mb) (Figure 3).

### mRNA expression profiling

**Figure 4:**
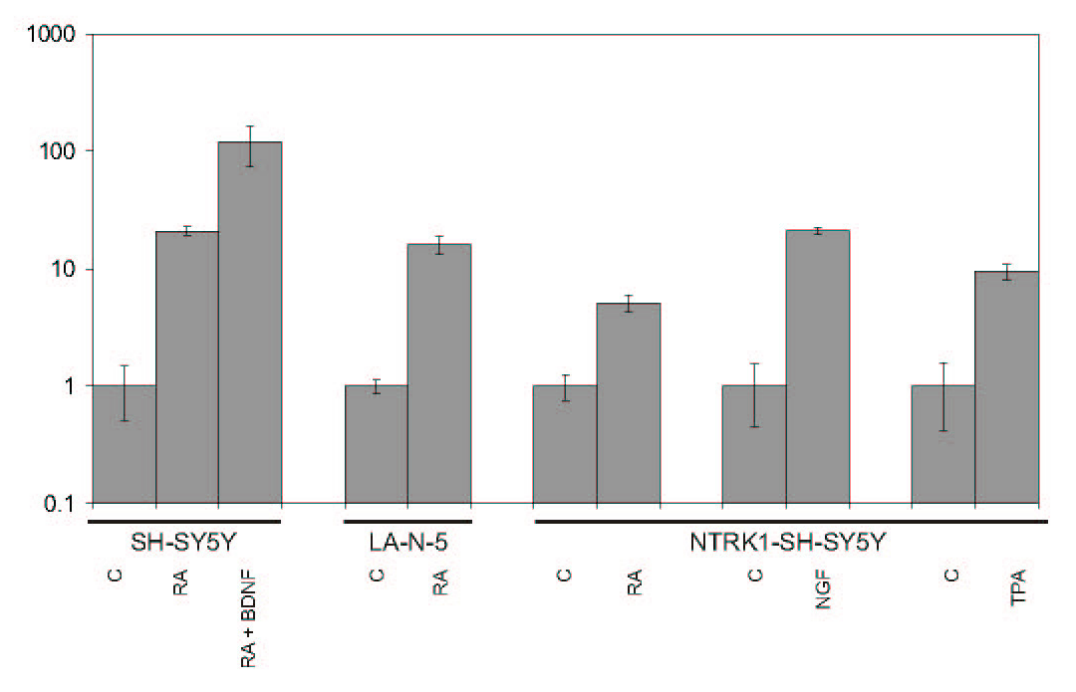
Fold induction of *HNT *mRNA expression (log scale, versus control cultures, C) in neuroblastoma cell lines SH-SY5Y, LA-N-5 and NTRK1-transfected SH-SY5Y after induction of differentiation with RA and BDNF (retinoic acid and brain-derived neurotrophic factor), NGF (nerve growth factor) and TPA (12-O-tetradecanoyl-phorbol-13-acetate).

As loss of 11q is a recurrent chromosomal aberration in a subgroup of advanced stage neuroblastomas, the 11q25->11qter region that is deleted in the MCH574c3 microcell hybrid is of particular interest. In an attempt to relate the observed morphology of induced neuronal differentiation to expression differences of genes located in this distal 11q25 segment, the expression of 6 known genes, i.e. *HNT*, *OPCML*, *JAM3*, *THY28*, *ACAD8 *and *B3GAT1 *was tested. Of particular interest are *HNT *and *B3GAT1*, because of their reported involvement in neurite outgrowth and neural crest development. We quantified the mRNA expression of these 6 genes in the microcell hybrids, the parental cell line and in neuroblastoma cell lines (SH-SY5Y, LA-N-5 and NTRK1 transfected SH-SY5Y) that were treated with inducers of differentiation [12-14]. While the expression of the genes is not significantly altered in the microcell hybrids compared to the parental cell line, the expression of *HNT *is significantly higher in different cell lines that are induced to differentiate (between 5 to 120 fold induction) (Figure 4).

## Discussion

In a search for candidate neuroblastoma genes located on chromosome arm 11q, we investigated microcell hybrids obtained by transfer of a normal chromosome 11 into NGP neuroblastoma cells with loss of 11q. Although initially designed as a control experiment, this transfer resulted in morphological changes in the obtained hybrids (without loss of tumorigenicity) and also yielded revertants after further culture [10]. The induced differentiation that was observed in all but one microcell hybrid is consistent with the presence of a neuroblastoma differentiation gene on chromosome 11. We thus anticipated that these hybrids might be of interest for functional mapping of the regions on chromosome 11 critically involved in neuroblastoma pathogenesis. To investigate this, we performed arrayCGH copy number analysis of these microcell hybrids. This allowed us to assess the status of the introduced (and parental) chromosomes 11 and to validate these hybrids as model system for further functional assays. The obtained results were surprising and puzzling. One particular microcell hybrid that did not show the expected differentiation features upon chromosome 11 transfer was shown to carry an 11q25->11qter deletion in the transferred chromosome. In addition we found that all microcell hybrid subclones presented with an 11pter->11p15.1 deletion, and that the MCH574c3 hybrids presented with an additional 11pter->11p13 deletion.

**Figure 5:**
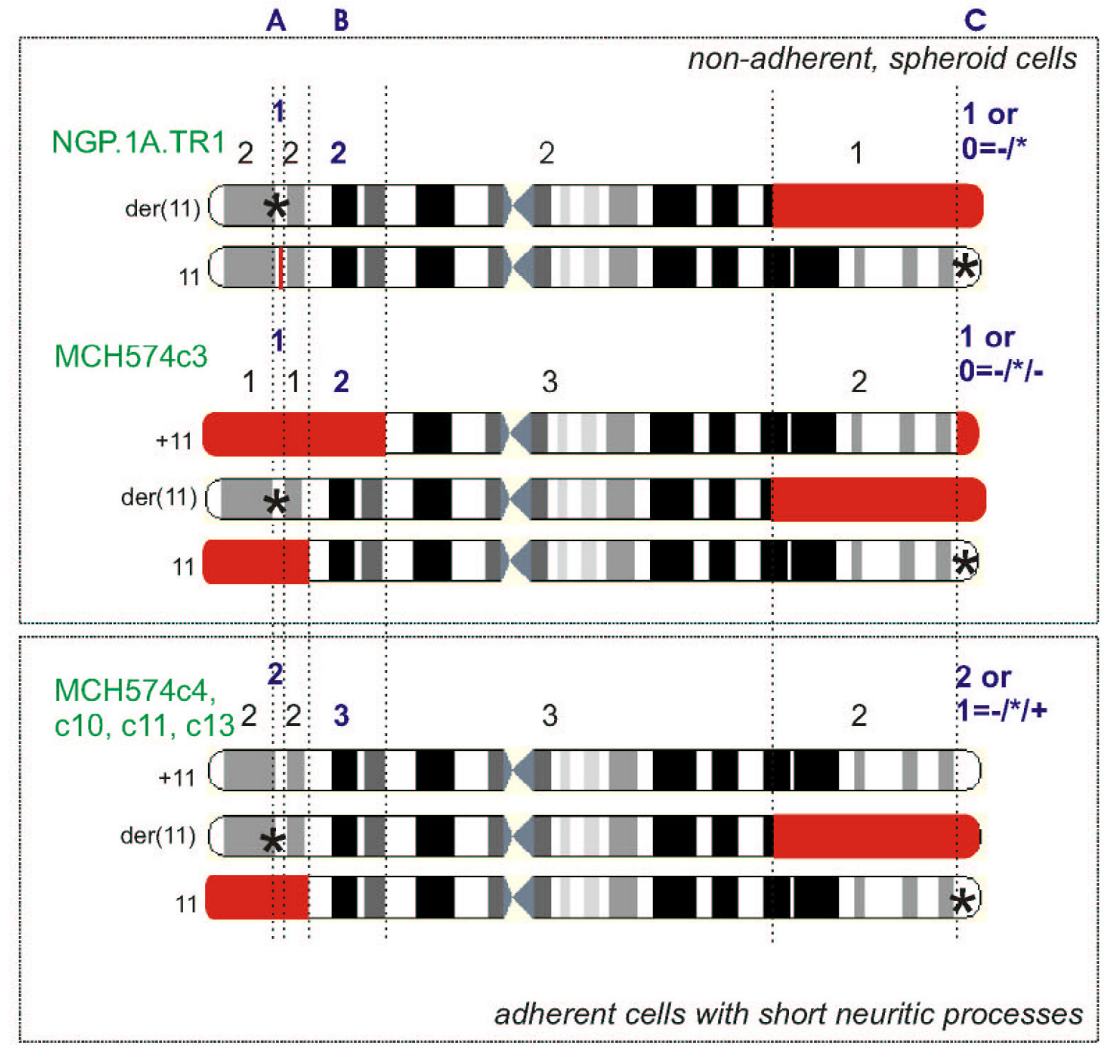
Regional copy numbers (deletion events are indicated in red) in cells with non-adherent, spheroid (parental) cell phenotype compared to cells with induced differentiation, demonstrating the three regions on chromosome 11 that may be involved in the phenotypic difference, i.e. a small region on 11p15.3 encompassing BAC clone RP11-51B23 (lost in NGP.1A.TR1) (A region), the 11p13->11p15.1 region (lost in MCH574c3 but not in the other MCH574 microcell hybrids) (B region) and the 11q25->11qter region (lost in MCH574c3) (C region) (* indicates the putative presence of a mutated gene).

In line with previous successful functional analyses of microcell hybrids [15], the responsible gene for the observed changes in cell morphology is assumed to be located in one of the chromosomal regions that show a different copy number in the microcell hybrid subclones with differentiation features (MCH574c4, c10, c11 and c13) compared to the non-adherent, spheroid cell phenotype of parental cell line NGP.1A.TR1 and microcell hybrid subclone MCH574c3. Based upon our findings three regions can be identified as candidate regions harbouring a putative differentiation gene: (1) the 11q25->11qter region (lost in MCH574c3), (2) the 11p13->11p15.1 region (lost in MCH574c3 but not in the other MCH574 subclones) and (3) a small region of at least 706 kb on 11p15.3 (lost in NGP.1A.TR1) (Figure 5).

As loss of distal 11q is a recurrent chromosomal aberration in *MYCN *single copy advanced stage neuroblastomas [3], we propose the 11q25->11qter chromosomal segment as the most likely candidate region for the presence of a differentiation gene. Despite efforts to define a shortest region of overlap (SRO) for 11q-loss in neuroblastoma by microsatellite heterozygosity mapping [7] and delineation of constitutional 11q-deletions [8,16], a consensus region for loss of 11q in neuroblastoma has not been defined thus far. In the light of the uncertainty of the boundaries of the 11q SRO, the 11q25->11qter region must be considered as potentially harbouring a neuroblastoma suppressor or differentiation gene. This region is present in two copies in microcell hybrid subclones MCH574c4, c10, c11 and c13 with differentiated morphology, but only in one copy in the non-adherent, spheroid cells from NGP.1A.TR1 and MCH574c3. Six known genes, i.e. *HNT*, *OPCML*, *JAM3*, *THY28*, *ACAD8 *and *B3GAT1 *are located in this distal 11q segment, of which two genes are of particular interest. *HNT *(neurotrimin) is reported to promote neurite outgrowth and adhesion [17]. *B3GAT1 *encodes for a protein that functions as the key enzyme in a glucuronyl transfer reaction during the biosynthesis of the carbohydrate epitope HNK1 (CD57) [18,19], which is a carbohydrate expressed in developmentally immature neural crest cells [20]. Interestingly, the expression of *HNT *is significantly increased in neuroblastoma cell lines that are induced to differentiate using RA (retinoic acid), RA plus BDNF (brain-derived neurotrophic factor), NGF (nerve growth factor) and TPA (12-O-tetradecanoyl-phorbol-13-acetate). However, *HNT *expression is not significantly different between the differentiated microcell hybrids and the parental cells. It is conceivable that the observed phenotypic changes are caused by small changes in expression that can not be reliably detected by Q-PCR. An alternative explanation is that the normal parental chromosome 11 harbours a mutated allele that is normally expressed at the mRNA level (Figure 5). Reintroduction of a wild type allele by chromosome transfer could repair the defect, leading to differentiation. This is in keeping with reversal to the non-adherent, spheroid morphology of the microcell hybrids that have lost the 11q25->11qter region of the transferred chromosome. Additional mutation, promoter hypermethylation and gene directed functional assays are needed to clarify which of the genes located within the deleted 11q25->11qter region are responsible for the differentiated phenotype.

While the 11q25 region is the best candidate region to harbour a differentiation gene, the observed deletions on the short arm of chromosome 11 may also account for the differentiated morphology. The observation of two independent deletion events along the distal part of chromosome arm 11p is suggestive for the involvement of this region. In particular, it is striking that all microcell hybrids in which chromosome 11 is transferred are characterised by the presence of an 11pter->11p15.1 deletion in the (prior to transfer) normal parental chromosome 11. This may either be the result of an early coincidental event during the transfer process, or indicative for a selection process against the presence of three copies of a growth suppressive gene in this region. The last hypothesis may be further supported by the presence of unbalanced 11p-deletions in 4% of neuroblastomas (14/394) [21,22].

Apart from highlighting at candidate 11q regions involved in neuroblastoma pathogenesis, this study clearly shows that it is important to monitor the transfer of the desired chromosome, as well as the genetic background of the cell line before and after chromosome transfer experiments. Selective pressure processes may occur during or after transfer of a chromosome, e.g. by chromosomal loss in order to maintain the viability of the microcell hybrids. Hence, detailed information on the chromosome copy number status before and after transfer is required in order to correlate phenotypic changes with chromosomal alteration. ArrayCGH has been proven to be a valuable screening method for evaluation of the chromosome alterations and for delineation of possible deletion events, allowing fine-mapping of the candidate regions that harbour candidate suppressor genes.

## Conclusion

Microsatellite marker heterozygosity analysis, FISH and (array)CGH based copy number in neuroblastoma tumour specimens and patients with constitutional deletions have thus far not identified a consensus SRO for 11q-deletion. Here, we present an alternative strategy to pinpoint chromosomal regions or genes that may be important in neuroblastoma pathogenesis. Chromosome 11 transfer, followed by phenotype scoring and high-resolution copy number analysis delineated putative regions on chromosome 11 involved in tumour differentiation. Further mutation and functional analyses are required to clarify the putative involvement of genes localised in these regions in neuroblastoma.

## Methods

### Cell lines

The parental cell line, NGP.1A.TR1, and the chromosome 11 microcell transfer derived subclones MCH574c3, c4, c10, c11 and c13 used in this study have been described previously [10].

Cell lines were cultured following standard procedures and were digitally photographed under an inverted (phase-contrast) microscope, pelleted, snap-frozen and stored at -80°C for further processing. DNA was isolated using the QIAamp DNA mini kit (Qiagen). RNA was isolated from the snap-frozen cell pellets using the RNeasy Mini kit (Qiagen) according to the manufacturer's guidelines, followed by RNase free DNase treatment on column (Qiagen).

Culture conditions and details regarding differentiation protocols are given in [12,13]. The NTRK1-transfected SH-SY5Y cells used, were SH-SY5Y/trkA, clone 6:2 described in [23].

### Phalloidin staining

Cell lines were fixed for 10 min in 4% paraformaldehyde/HEPES on ice. The excess of aldehydes is quenched for 5 min in 50 mM NH_4_Cl. After washing twice for 5 min in 1x PBS, extraction is performed for 5 min in acetone (20°C). The cells are washed again twice for 5 min in 1x PBS, followed by blocking in 0.2% Fish Skin Gelatine (FSG, Sigma)/PBS. During 60 min cells are incubated with Alexa 594-phalloidin (1 unit per section), dissolved in 0.2%FSG/PBS at 37°C. Cells are washed twice for 5 min in 1x PBS; nuclei are stained for 1 min with DAPI; sections are washed with 1x PBS and mounted in Vectashield.

### ArrayCGH

ArrayCGH using 1 Mb BAC arrays was performed once for NGP.1A.TR1, MCH574c3 and MCH574c10 cells with normal female DNA as control. In addition, subclones MCH574c3 and MCH574c10 were hybridised to the same arrays with NGP.1A.TR1 DNA as control. Hybridisation of cell line and control DNA to the array was performed as described [24]. Using our in-house developed analysis and visualisation software, arrayCGHbase, data were normalised to the median ratio, and replicate median ratio profiles visualised    [29].

### FISH and microsatellite marker analysis

BAC clones and microsatellite markers were selected based on their chromosomal position using the Ensembl genome browser , the UCSC human genome browser (July 2003 freeze, ) or the Genome Database . Labelling and FISH (fluorescence in situ hybridisation) was performed as described [25]. Experimental conditions for the fluorescent based microsatellite screening can be obtained from the authors upon request.

### Real-time quantitative RT-PCR based mRNA expression profiling

Primers were designed using Primer Express v2.0 (Applied Biosystems). Primer sequences are available in the public RTPrimerDB database : *HNT *(1078), *OPCML *(1079), *JAM3 *(1080), *THY28 *(1084), *ACAD8 *(1081), *B3GAT1 *(1082), *HPRT1 *(5), *UBC *(8) and *GAPD *(3) [26]. Relative expression levels were determined using an optimized two-step SYBR Green I RT-PCR assay [27]. PCR reagents were obtained from Eurogentec as SYBR Green I core reagents, prepared as 2x mastermixes, stored at -20°C and used according to the manufacturer's instructions. Reactions were run on an ABI5700 (Applied Biosystems). The comparative C_T _method was used for quantification. Gene expression levels were normalized using the geometric mean of the 3 most stable internal control genes in neuroblastoma (i.e. *UBC*, *HPRT1 *and *GAPD*) as reported previously [28].

## Abbreviations

arrayCGH = microarray based comparative genomic hybridisation

FISH = fluorescence *in situ *hybridisation

MMCT = microcell mediated chromosome transfer

SRO = shortest region of overlap

## Authors' contributions

KDP supervised the culturing of the microcell hybrids that were produced by SB, carried out the microsatellite marker analysis and drafted the manuscript. PC performed the arrayCGH hybridisations, under the supervision of HF and NC. KDP and BM analysed the arrayCGH data. WW performed the phalloidin staining and NVR evaluated the FISH results. NY performed real-time quantitative PCR. Neuroblastoma cells were induced to differentiate by AE under the supervision of SP. JV and FS participated in the study design and coordination, and were the final editors of the manuscript.
